# Potential Therapies to Protect the Aging Heart Against Ischemia/Reperfusion Injury

**DOI:** 10.3389/fcvm.2021.770421

**Published:** 2021-11-19

**Authors:** Magda C. Díaz-Vesga, Úrsula Zúñiga-Cuevas, Andrés Ramírez-Reyes, Nicolas Herrera-Zelada, Iván Palomo, Roberto Bravo-Sagua, Jaime A. Riquelme

**Affiliations:** ^1^Programa de Fisiología y Biofísica, Instituto de Ciencias Biomédicas (ICBM), Facultad de Medicina, Universidad de Chile, Santiago, Chile; ^2^Grupo de Investigación en Ciencias Básicas y Clínicas de la Salud, Pontificia Universidad Javeriana de Cali, Cali, Colombia; ^3^Advanced Center for Chronic Disease (ACCDiS), Facultad de Ciencias Químicas y Farmacéuticas, Facultad de Medicina, Universidad de Chile, Santiago, Chile; ^4^Thrombosis Research Center, Faculty of Health Sciences, Universidad de Talca, Talca, Chile; ^5^Interuniversity Center for Healthy Aging, Chile; ^6^Instituto de Nutrición y Tecnología de los Alimentos (INTA), Universidad de Chile, Santiago, Chile

**Keywords:** cardioprotection, aging, ischemia/reperfusion injury, mitochondria, senolytics

## Abstract

Despite important advances in the treatment of myocardial infarction that have significantly reduced mortality, there is still an unmet need to limit the infarct size after reperfusion injury in order to prevent the onset and severity of heart failure. Multiple cardioprotective maneuvers, therapeutic targets, peptides and drugs have been developed to effectively protect the myocardium from reperfusion-induced cell death in preclinical studies. Nonetheless, the translation of these therapies from laboratory to clinical contexts has been quite challenging. Comorbidities, comedications or inadequate ischemia/reperfusion experimental models are clearly identified variables that need to be accounted for in order to achieve effective cardioprotection studies. The aging heart is characterized by altered proteostasis, DNA instability, epigenetic changes, among others. A vast number of studies has shown that multiple therapeutic strategies, such as ischemic conditioning phenomena and protective drugs are unable to protect the aged heart from myocardial infarction. In this Mini-Review, we will provide an updated state of the art concerning potential new cardioprotective strategies targeting the aging heart.

## Introduction

Although the available treatments of acute myocardial infarction (MI) have improved in the past decades, the consequent heart failure is consistently rising and thus, effective protection of the heart to preserve ventricular function and reduce myocardial remodeling is still an unmet need (1). Several years of research have established that cardiomyocytes have signaling pathways that, upon activation, can reduce the damage elicited by ischemia/reperfusion (I/R) injury, such as the Reperfusion Injury Salvage Kinases (RISK) and the Survivor Activating Enhancement Factor (SAFE) (2–4). There are numerous cardioprotective strategies that can be induced by different ischemic conditioning procedures and drugs with successful results in preclinical studies (5). Nonetheless, translation of cardioprotection from bench to bedside has been highly challenging (6). Remote ischemic conditioning (RIC) is an important example of this. This cardioprotective maneuver has been thoroughly established as a potent therapeutic strategy in multiple preclinical studies, as well as small clinical trials (5). However, the CONDI-2/ERIC-PPCI trial showed solid evidence that RIC does not provide protection after 1 year in patients with ST-elevation myocardial infarction treated with primary percutaneous coronary intervention (7). It is currently proposed that this discrepancy between preclinical and clinical settings may be attributed to factors such as comorbidities and comedications (8), as well as sex and age (9, 10).

In the context of myocardial infarction and cardioprotection, aging is increasingly attracting attention, due to its rising prevalence. Over the past decades, life expectancy has been steadily increasing (11). It has been estimated that by 2050, a quarter of the global population will be over 65 years old, with the exception of Africa (12). Importantly, although life expectancy has increased, disease-free lifespan has not been markedly increased as compared with lifespan, thereby increasing the burden of age-related diseases, such as cancer, neurodegenerative and cardiovascular diseases (11). Indeed, aging is considered a significant risk factor for cardiovascular diseases (13, 14) and particularly, for MI (15). Accordingly, chronic low-grade inflammation associated with aging is known to promote endothelial and vascular smooth muscle dysfunction, thus exacerbating atherosclerosis and increasing the risk of plaque rupture and thrombus formation (16).

The hallmarks of myocardial aging may account for the reduced tolerance against myocardial I/R injury in preclinical studies (17) and thus, its understanding may enable the development of new and effective therapies to reduce cardiac damage after MI in the context of aging. This mini review aims to discuss potential cardioprotective approaches in the aging heart such as the use of senolytics, as well as therapeutic strategies that aim to decrease mitochondrial damage and reduce inflammation.

## The Aging Cardiomyocyte

**Figure 1:**
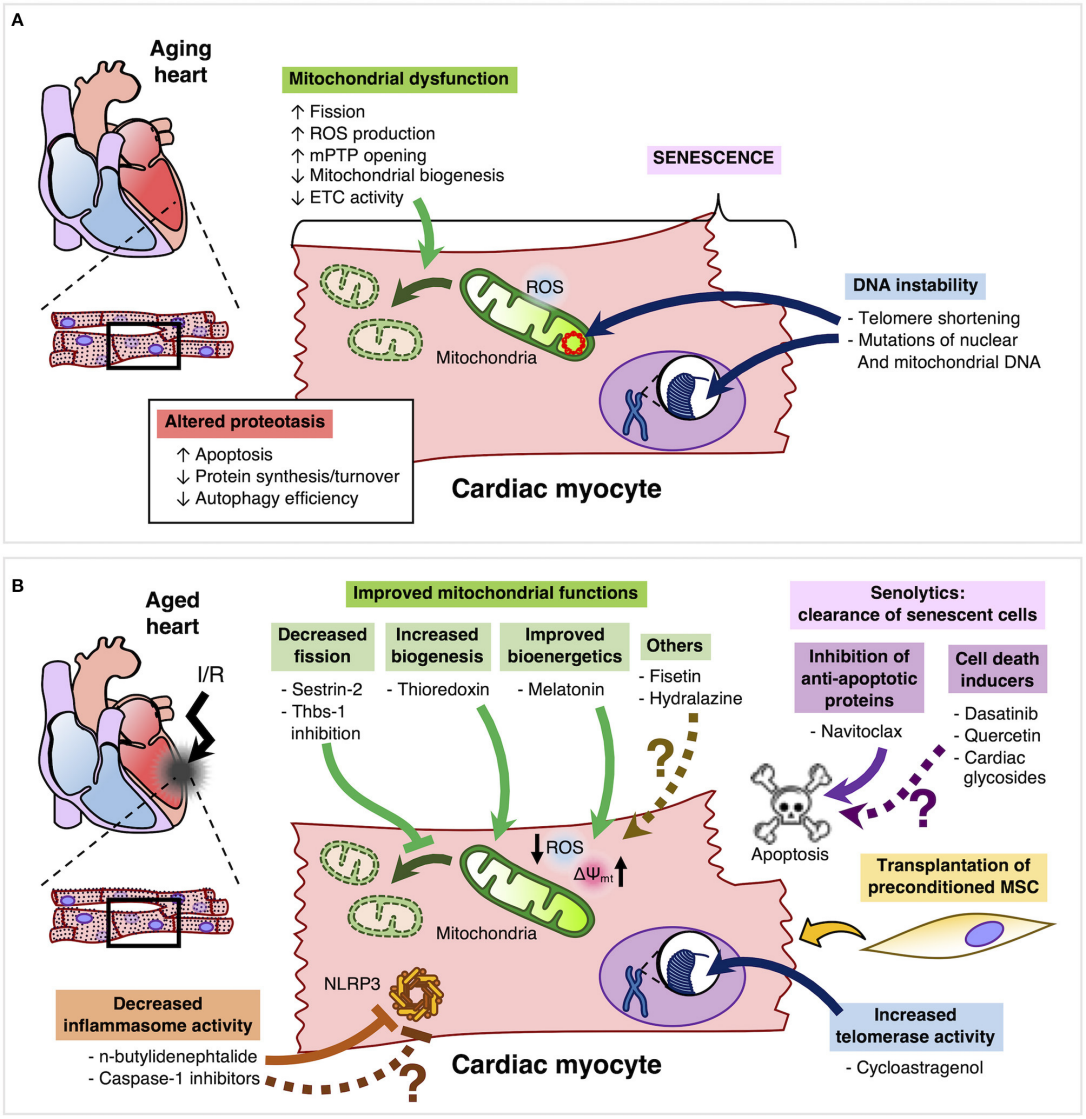
**(A)** Aging cardiomyocytes are mainly characterized by mitochondrial dysfunction, senescence, altered proteostasis and DNA instability. **(B)** Improvement of mitochondrial dysfunction in aged cardiomyocytes may be achieved by inhibition of Thbs-1 and Sestrin-2, which can decrease Drp-1-mediated mitochondrial fission. Moreover, thioredoxin can promote mitochondrial biogenesis and melatonin can improve bioenergetics by reducing ROS production and improving mitochondrial membrane potential. Other compounds such as Fisetin and hydralazine can confer cardioprotection by reducing mitochondrial dysfunction, but whether these compounds can effectively limit the infarct size and improve left ventricular function of the aging heart remains to be elucidated. Senolytics can clear senescent cells by inhibiting anti-apoptotic proteins. However, only Navitoclax has been tested in the aged heart. However, other senolytics such as Dasatinib, Quercetin, and cardiac glycosides are yet to be evaluated in the settings of cardioprotection and aging. Furthermore, activation of telomerase in order to preserve telomere length and integrity is a promising candidate for cardioprotection in the aging heart. Another emergent target is to inhibit the inflammasome by compounds such as n-butylidenephtalide. Finally, the transplantation of preconditioned mesenchymal cells may have an important therapeutic role in the protection of the aged myocardium.

One of the key features of the aging heart is mitochondrial dysfunction, characterized by increased mitochondrial fragmentation, ROS production and mitochondrial permeability transition pore opening, as well as reduced mitochondrial biogenesis and electron transport chain activity (17–19). Furthermore, senescent cardiomyocytes also undergo DNA instability, mostly related with telomere shortening and mutations of nuclear and mitochondrial DNA (20). Altered proteostasis is another important feature of aging cardiomyocytes, whereby a reduction in protein synthesis/turnover is associated with a decreased efficiency of the ubiquitin-proteasome system and autophagy (21). The main features of the aged cardiomyocyte are summarized in Figure 1A. In addition, Ruiz-Meana et al. provide an in depth analysis and discussion of the aged cardiomyocyte in the context of cardioprotection (17).

## Potential Cardioprotective Therapies in the Aged Heart

### Senolytics: Cardioprotective Potential in the Aged Heart

Senescent cells increase in aged tissues, which has been associated with the progression of age-related diseases (22). In this context, senescence markers are augmented in aged cardiomyocytes (23), which has been linked to higher risk of cardiovascular diseases (24). Senolytics are agents that can selectively target pro-survival proteins of senescent cells, inducing cell death. Regarding the heart, there are three major senolytics that have been widely studied *in vivo* and *in vitro*; Dasatinib, Quercetin, and Navitoclax (24). These senolytics have been shown to improve vascular function (25, 26). Importantly, a study performed by Walaszczyk et al. showed that oral administration of navitoclax to aged mice before *in vivo* myocardial infarction reduced morality, as well as age-related myocardial remodeling and improved left ventricular function (27).

Despite the beneficial effects of senolytics on a preclinical level, there are only two clinical studies showing that these drugs can decrease senescent cells in humans (28, 29). Justice et al., showed that a combination of Dasatinib + Quercetin in patients with idiopathic pulmonary fibrosis can improve physical function (29), while Hickson et al. observed that administration of Dasatinib + Quercetin in patients with diabetic kidney disease elicited a decrease in senescent cells of adipose tissue (28). Despite showing efficacy in treatment of myocardial infarction in the aged heart, Navitoclax has yet to be tested in humans with cardiovascular diseases. Overall, senolytics are a promising pharmacological approach, which appears to effectively reduce senescent cells in humans, but additional preclinical evidence is warranted before they can be translated into the clinical setting of cardioprotection in the elder population.

Currently, there are ongoing efforts to identify new or old compounds that can decrease the number of senescent cells (30). For instance, cardiac glycosides (CGs), have been recently identified as potential senolytic compounds that can successfully decrease the number of senescent cells in the context of cancer in old mice (31) and senescence-induced lung fibrosis (32) in mice. Both of these studies showed that the senolytic effect exerted by CGs depends on the inhibition of Na^+^/K^+^ ATPase pump (31, 32). Nonetheless, despite the novelty of using these old drugs to clear senescent cells, there are currently no studies showing whether CGs can protect the adult or aged myocardium from I/R injury and thus, this research field is still in preliminary stages in the context of cardioprotection. Moreover, Ouabain is a cardiac glycoside that has been shown to impair growth of proliferative cells in human epithelial cells in a context of oncogene-induced senescence (33) and thus, the specificity of these drugs for senescent cells must be thoroughly evaluated before considering their use in MI.

Recently, an interesting non-pharmacological approach has been investigated. Amor et al. showed that the urokinase-type plasminogen activator receptor (uPAR)-a cell-surface marker that is overexpressed in senescent cells- is a target for chimeric antigen receptor (CAR) T cells, which can clear senescent cells in mice both *in vitro* and *in vivo* in the context of lung adenocarcinoma and liver fibrosis (34), thus providing a promising proof of concept of therapeutic agents for the treatment of senescence-associated diseases, such as MI of the aging heart. However, concerns have been raised over the adverse effects of this therapy. The most frequently reported side effects are cytokine release syndrome and neurotoxicity, but cardiovascular toxic effects, such as systolic dysfunction, arrhythmias, or hypotension have also been reported and thereby, a thorough assessment of cardiac imaging and evaluation of reliable biomarkers is needed to identify at-risk population and establish the safety of this therapy (35).

### Role of Mitochondria in Cardioprotection of the Aged Heart

The mitochondria have been identified as an important target to reduce myocardial ischemia/reperfusion injury in the aged heart (36). The mitochondria in aging cardiomyocytes shows elevated ROS production, higher fragmentation and reduced biogenesis, thus producing mitochondrial dysfunction, which can contribute to the increased susceptibility of the aged heart to ischemic injury (17). Therefore, therapies targeting the mitochondria are an attractive area of research in cardioprotection.

It has been reported that Thrombospondin-1 (Thbs-1), which is known to induce ROS production after I/R, regulates Drp-1 dependent mitochondrial fission after MI in both young and old mice (37). Thus, inhibition of Thbs-1 levels after I/R may reduce mitochondrial fission and ROS production, thereby protecting the aging heart against I/R injury. Furthermore, it has been shown that overexpression of thioredoxin -a cytosolic redox protein with reducing properties- in aged mice subjected to *in vivo* I/R, improved mitochondrial function and biogenesis, as well as reducing the infarct size and left ventricular dysfunction (38). Another potential target for cardioprotection is Sestrin2. This protein is induced by stress and forms a complex with AMP-activated protein kinase (AMPK), thus promoting autophagy. Importantly, its cellular levels drop with age, and it's been shown that a Sestrin2-KO promotes mitochondrial fission and damage after myocardial I/R in mice (39). Moreover, overexpression of Sestrin2 in aged mice hearts improved the heart's response to MI (39). Finally, melatonin is a molecule secreted by the pineal gland in humans. It exerts antioxidant and anti-inflammatory properties that may play a cardioprotective role (40). A study in aged rats showed that administration of melatonin and nicotinamide mononucleotide reduces the infarct size and improves left ventricular function after I/R injury by reducing mitochondrial ROS and improving mitochondrial membrane potential (41). Moreover, it has been suggested that melatonin protects against I/R injury via the RISK and SAFE pathways (42). Interestingly, a small clinical trial has provided proof of concept that the timing of administration of melatonin in infarcted patients undergoing percutaneous coronary intervention is key for its effectiveness (43). However, this observation needs to be confirmed in large randomized controlled clinical trials.

There are multiple strategies and treatments that target the mitochondria to achieve cardioprotection that have not been yet studied in the context of aging. We will discuss two of them: fisetin and hydralazine. Fisetin is a flavonoid found in many fruits with senolytic properties (44). Recently it's been shown that it can also confer protection in I/R settings. A preliminary study showed that fisetin can inhibit H9c2 cardiomyoblast apoptosis after hypoxia/reoxygenation, as well as to reduce mitochondrial ROS production and inhibit the activation of caspases 3 and 9 (45). In addition, adult rats pre-treated with fisetin and subjected to *ex vivo* I/R showed a reduced infarct size, reduced mitochondrial oxidative stress and improved mitochondrial structure and function by a mechanism that appears to involve inhibition of GSK3β activity (46). Hydralazin, is an FDA approved drug currently used as an anti-hypertensive agent and in the treatment of chronic heart failure. Recently, it has been described that it can confer cardioprotection in mice using *in vivo, ex vivo* and *in vitro* experimental models of I/R injury via inhibition of Drp-1-mediated mitochondrial fission (47). These two molecules have in common their capacity to protect the heart from I/R injury by improving mitochondrial function and thus, these therapeutic agents are promising candidates to protect the aging heart from MI. Therefore, future studies should explore this possibility.

### Inflammasome: Importance of NLPR3 in Aging

During MI, a pathogen/antigen-independent inflammatory response, known as sterile inflammation, takes place (48). Due to the rupture in the cellular structure that occurs during MI, damage-associated molecular patterns (DAMPS) mediators are released and are recognized by pattern recognition receptors (PRRs), which in turn mediate the initiation of the inflammatory response (49). NLPR3 inflammasome is a multiprotein complex formed by the activation of PRRs, thereby increasing the production and release of proinflammatory cytokines via activation of caspase-1 (50). NLPR3 has attracted attention as a mediator of damage produced by I/R injury (51). Mastracola et al. tested the role of NLPR3 in C57B1 male mice fed with a high fat high fructose diet (HFHF) using an *ex vivo* I/R model. The authors found that the HFHF diet upregulated NLPR3 protein content, and this elevation downregulated the RISK/HIF-2alpha pathways (51–53). Furthermore, NLPR3 has also been proposed as a target in the aging myocardium. Lee et al. has explored this hypothesis in a recent study where they use *n*-butylidenephthalide as a preconditioning agent and tested the capacity of human adipose–derived stem cell (hADSC) engraftment in the recovery of infarcted myocardium of young and old Wistar rats (54). The study showed NLPR3 activity and ROS production was significantly increased in old rats after MI, as compared with young rats. Transplantation of hADSC reduced NLPR3 activation and ROS levels in both young and old rats, but this response was significantly more effective in young rats. The authors discovered that the NLPR3 inflammasome mediates the difference of the response to the engraftments in old and young rats. They also reported that n-butylidenephthalide reversed the complex microenvironment that impedes engraftment success (54).

Interestingly, pharmacological inhibition of caspase-1 reduced the infarct size in isolated rat hearts (55). Also, caspase-1 inhibition was also shown to provide additional protection when combined with remote ischemic preconditioning in rats subjected to *in vivo* myocardial infarction (56). This study is particularly interesting, since these experiments were performed using a co-medication model, consisting of co-administration with an opioid agonist, heparin and a platelet-inhibitor to mimic a clinical setting, thereby highlighting the translational value of this cardioprotective therapy (56). This promising evidence merits further research to evaluate whether this approach may have a significant impact in the cardioprotection of the aged heart.

### Telomerase Activity in Aging and Cardioprotection

Telomeres are repeated hexanucleotide sequences at the end of eukaryotic chromosomes. Their presence is associated with DNA protection during cell division. Division of the cell as well as oxidative stress shortens these structures, leading the cell to a senescent state or apoptosis (57). Telomere length has been associated with coronary artery disease and therefore, it has been proposed as a biomarker for cardiovascular diseases (58). Telomerase is a key regulator of telomere length and integrity (20) and as such, has gained attention for its potential benefits in age-related cardiovascular diseases. For instance, absence of telomerase has been associated with increased susceptibility to ischemic injury (59). By the same token, overexpression of telomerase can confer cardioprotection in mice hearts (60). Furthermore, overexpression of growth differentiation factor 11 (GDF11) in mice subjected to *in vivo* I/R injury showed a reduced infarct size, activation of telomerase, longer telomeres and increased mitochondrial biogenesis (61). In a clinical context, Gupta et al. have published a pilot study that proposes telomere length as a screening tool for patients with acute myocardial infarction (62). Their study revealed that telomere length was reduced in young patients without risk factors who underwent MI, as compared with young patients without MI (62). Moreover, Maier et al. have published the design of a pilot trial that uses the telomerase activator TA-65MD, which is a purified and encapsulated form of cycloastragenol, in elderly patients with acute coronary syndrome (63). The aim of this study is to assess whether this therapy can reverse inmmunosenescence, which is associated to the pathophysiological progression of coronary artery disease. The results to this trial are scheduled to be published in 2021 (63). Although more preclinical data is required to accurately assess whether this therapeutic strategy can reduce cardiac damage in aging, telomerase appears to be an essential player in cardioprotection.

### Other Potential Therapies to Protect the Aged Myocardium From I/R Injury

In addition to the aforementioned strategies, there are other approaches that may wield cardioprotective effects in aging conditions. An interesting study has suggested that necroptosis may contribute to ischemic susceptibility in the aging heart and that metformin reduces necroptosis elicited by alterations of autophagy during myocardial aging (64). Another potential therapeutic target may be the JAK/STAT pathway. It has been reported that inhibition of JAK in old mice has been linked with reduced senescence-associated secretory phenotype and frailty (65). Moreover, deletion of Kcne4 in old mice has been found to sex-specifically induce arrhythmias by a mechanism that involves defective signaling of the RISK/SAFE pathway mediated by testosterone (66). Furthermore, it has been observed that the JAK/STAT pathway is activated in aging humans, which is associated with chronic inflammation and increased cardiovascular risk (67). However, whether targeting the JAK/STAT pathway may restore the effectiveness of protective strategies in the context of MI remains to be elucidated.

Aging has also been described to alter mesenchymal stem cell function, which impairs their cardioprotective effects (68). It has been recently identified that miR-155-5p can regulate mesenchymal stem cell senescence (69). This study demonstrated that inhibition of miR-155-5p reduced senescence in aged mesenchymal stem cell, thus improving their ability to protect the aged heart from MI in mice (69).

Finally, a study by Crewe et al. revealed that adipocytes can secrete small extracellular vesicles that can transport damaged mitochondria, which can precondition the heart by inducing a short period of mitochondrial oxidative stress, which eventually leads to an antioxidant effect that can effectively protect the heart from MI (70). While this effect has not been evaluated in the aged heart, protection elicited by transport of mitochondria by small extracellular vesicles via hormesis may be an effective therapy, given the importance of this organelle in the aging myocardium.

### Conclusions and Future Perspectives

The aged heart is more susceptible to I/R injury. Moreover, multiple cardioprotective maneuvers have been shown to be ineffective under these conditions, highlighting the need to develop therapies that can provide clinical efficacy in the reduction of infarct size. Clearance of senescent cells by the senolytic drug navitoclax has shown clear protection of aged hearts from MI, but the current evidence is still preliminary and more senolytics need to be tested to establish the real potential of these agents. Moreover, the clinical evidence regarding the use of these drugs is still in early stages, especially since there are no studies testing their effects in MI patients. Cardioprotective approaches targeting the mitochondria in the aged heart are promising, although the current evidence is mainly centered in preclinical studies, suggesting that the translational value of these therapies remains to be tested in clinical settings. Similarly, targeting of the inflammasome or telomerase activity are research areas of interest, but further studies are certainly required to evaluate whether these therapeutic strategies can be translated from bench to bedside. Currently, cardioprotection in aging can be tested using primary cells, *ex vivo* and *in vivo* animal models to evaluate the effect of protective drugs or conditioning maneuvers (17). The use of cell lines may be useful to assess stress-induced premature senescence and thus generate a preliminary proof-of-concept regarding a potential senolytic therapy, but while this allows to avoid the use of animals, this approach only elicits a senescence-like phenotype (71). Therefore, this model may have a low translational value. Regarding cardioprotection research, preclinical models are solid in the early stages, but clinical studies are strong in the follow-up process (72). In this context, Heusch has proposed to use integrative large animal models and study the whole process: from acute myocardial infarction to a 12 months follow-up (72) and this idea is certainly valid for the study of cardioprotection in aging as well. Furthermore, it's crucial to consider the importance of generating reliable evidence to drive cardioprotection research forward by adhering to proposed guidelines to ensure rigor and reproducibility, such as the ones presented by Bøtker et al. (73). Finally, while the potential therapies we have reviewed are promising (Figure 1B), it is important to acknowledge a lack of studies in this field. For instance, while aging can impair the cardioprotective effect of conditioning strategies in preclinical studies (8) a retrospective study showed that age did not disrupt cardioprotection in coronary artery bypass graft patients with or without RIC (74). Thus, while the evidence showing aging as a confounding factor in cardioprotection is thorough, clinical evidence is mainly obtained from retrospective secondary analyses (8), highlighting the distance between bench and bedside, which is barrier that needs to be overcome to protect the elderly from myocardial infarction.

## Author Contributions

MD-V, ÚZ-C, AR-R, NH-Z, IP, RB-S, and JR participated in the conception and design of the manuscript and made significant contributions to the analysis of the evidence discussed. All authors wrote the manuscript and gave the final approval of the submitted manuscript.

## Funding

This work was supported by CONICYT FONDAP grant 15130011 (to JR and RB-S), FONDECYT Iniciación 11181000 (to JR) and 11201267 (to RB-S), Subvención a la Instalación PAI 77170004 (to RB-S), Universidad de Chile grants ABCvital 02-2018 and U-Inicia UI-006/19 (to RB-S), and ANID fellowship 21191341 (to MD-V).

## Conflict of Interest

The authors declare that the research was conducted in the absence of any commercial or financial relationships that could be construed as a potential conflict of interest.

## Publisher's Note

All claims expressed in this article are solely those of the authors and do not necessarily represent those of their affiliated organizations, or those of the publisher, the editors and the reviewers. Any product that may be evaluated in this article, or claim that may be made by its manufacturer, is not guaranteed or endorsed by the publisher.
